# Novel *KCND3* Variant Underlying Nonprogressive Congenital Ataxia or SCA19/22 Disrupt K_V_4.3 Protein Expression and K+ Currents with Variable Effects on Channel Properties

**DOI:** 10.3390/ijms22094986

**Published:** 2021-05-07

**Authors:** Ginevra Zanni, Cheng-Tsung Hsiao, Ssu-Ju Fu, Chih-Yung Tang, Alessandro Capuano, Luca Bosco, Federica Graziola, Emanuele Bellacchio, Serenella Servidei, Guido Primiano, Bing-Wen Soong, Chung-Jiuan Jeng

**Affiliations:** 1 Department of Neurosciences, Unit of Neuromuscular and Neurodegenerative Disorders, B. Gesù Children’s Hospital, IRCCS, 00146 Rome, Italy; luca.bosco@opbg.net; 2 Department of Physiology, College of Medicine, National Taiwan University, Taipei 100, Taiwan; hsiaoct26@gmail.com (C.-T.H.); d01441001@ntu.edu.tw (S.-J.F.); tang@ntu.edu.tw (C.-Y.T.); 3 Department of Neurology, School of Medicine, National Yang Ming Chiao Tung University College of Medicine, Taipei 112, Taiwan; bwsoong@gmail.com; 4 Department of Neurology, Taipei Veterans General Hospital, Taipei 112, Taiwan; 5 Institute of Anatomy and Cell Biology, College of Medicine, National Yang Ming Chiao Tung University, Taipei 112, Taiwan; 6 Unit of Neurology, B. Gesù Children’s Hospital, IRCCS, 00146 Rome, Italy; alessandro.capuano@opbg.net (A.C.); federica.graziola@opbg.net (F.G.); 7 Department of Sciences, University Roma Tre, Viale Marconi 446, 00146 Rome, Italy; 8 Genetics and Rare Diseases Division, Bambino Gesù Children’s Hospital, IRCCS, 00146 Rome, Italy; emanuele.bellacchio@opbg.net; 9 Department of Neurosciences, Università Cattolica del S. Cuore, 00168 Rome, Italy; serenella.servidei@unicatt.it (S.S.); guido.primiano@gmail.com (G.P.); 10 Fondazione Policlinico Universitario A.Gemelli, IRCCS, 00168 Rome, Italy; 11 Department of Neurology, Shuang Ho Hospital, Taipei Neuroscience Institute, Taipei Medical University, New Taipei City 235, Taiwan

**Keywords:** *KCND3*, Spinocerebellar Ataxia SCA19/22, Congenital Ataxia, K_V_4.3

## Abstract

*KCND3* encodes the voltage-gated potassium channel K_V_4.3 that is highly expressed in the cerebellum, where it regulates dendritic excitability and calcium influx. Loss-of-function K_V_4.3 mutations have been associated with dominant spinocerebellar ataxia (SCA19/22). By targeted NGS sequencing, we identified two novel *KCND3* missense variants of the K_V_4.3 channel: p.S347W identified in a patient with adult-onset pure cerebellar syndrome and p.W359G detected in a child with congenital nonprogressive ataxia. Neuroimaging showed mild cerebellar atrophy in both patients. We performed a two-electrode voltage-clamp recording of K_V_4.3 currents in Xenopus oocytes: both the p.G345V (previously reported in a SCA19/22 family) and p.S347W mutants exhibited reduced peak currents by 50%, while no K+ current was detectable for the p.W359G mutant. We assessed the effect of the mutations on channel gating by measuring steady-state voltage-dependent activation and inactivation properties: no significant alterations were detected in p.G345V and p.S347W disease-associated variants, compared to controls. K_V_4.3 expression studies in HEK293T cells showed 53% (p.G345V), 45% (p.S347W) and 75% (p.W359G) reductions in mutant protein levels compared with the wildtype. The present study broadens the spectrum of the known phenotypes and identifies additional variants for *KCND3*-related disorders, outlining the importance of SCA gene screening in early-onset and congenital ataxia.

## 1. Introduction

Loss of function variants in the potassium voltage-gated channel subfamily D member 3 (*KCND3*/K_V_4.3) have been originally associated with spinocerebellar ataxia SCA19/22 (OMIM# 607346), a rare inherited neurodegenerative disorder characterized by slowly progressive ataxia, and a variable occurrence of cognitive impairment, signs of frontal lobe dysfunction, postural head tremor, seizures, pyramidal signs and neuropathy [1,2]. Only a few cases of *KCND3*-related nonprogressive congenital ataxia have been reported [3,4]. K_V_4.3 rapidly activates and inactivates in response to membrane depolarization, contributing to the neuronal subthreshold A-type potassium currents and controlling the action potential repolarization and frequency, and thus neuronal excitability [5]. K_V_4.3 is the only channel molecule showing anterior–posterior compartmentalization in the granular layer of the mammalian cerebellum and is highly expressed in migrating Purkinje cells during cerebellar development [6]. K_V_4.3 subunits co-assemble into functional tetrameric channels to form the pore domain (transmembrane helices S5–S6) surrounded by the S1–S4 segments which form the voltage-sensor domain [7]. Pore-forming voltage-gated α-subunits (Shal/K_V_4) associate with auxiliary β-subunits K_V_ Channel Interacting Proteins (KChIPs) which can modify protein trafficking, channel expression and activity [8]. We analyzed the clinical and molecular features of three *KCND3* variants, associated with SCA19/22 or congenital ataxia, underscoring the pathogenic role of impaired protein expression and reduced K^+^ currents with variable effects on channel gating in the dominant negative effect of different types of ataxia causing K_V_4.3 mutants.

## 2. Results

### 2.1. Clinical Description

The clinical features of the patients are summarized in Table 1.

**Figure 1:**
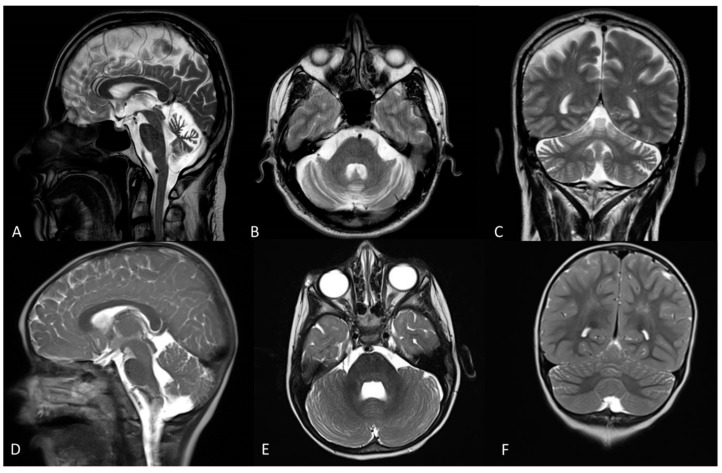
Brain MRI of patient 1 performed at 57 years old. T1-weighted midsagittal (**A**) axial (**B**) and coronal (**C**) sections showing atrophy of the cerebellar vermis and hemispheres. Brain MRI of patient 2 performed at age 2 years. T1-weighted midsagittal (**D**) axial (**E**) and coronal (**F**) sections showing mild atrophy of the superior cerebellar vermis and hemispheres.

Patient 1 is a 57-year-old man born at term from nonconsanguineous healthy parents. The early developmental milestones were normal. He had a progressive gait disorder from the age of 45 and subsequent clear limb and gait ataxia, dysarthria and dysphagia but no nystagmus (SARA score: 15) (Supplemental Video). His cardiological, biochemical and metabolic workup were normal. Brain and spinal MRI images showed atrophy of the cerebellar vermis and hemispheres (Figure 1a–c). Nerve conduction studies and electroencephalogram (EEG) were normal. Overall, the disease course was slowly progressive.

Patient 2 is a 7-year-old girl born at term from nonconsanguineous healthy parents. Developmental milestones were delayed: she was able to sit at 8 months and she walked independently at the age of 17 months. Gait ataxia and mild dysmetria with slow saccades were observed at the age of 1.5 years. Language delay, attention deficit, hyperactivity and oppositional behavior were present. EEG and cardiac evaluations were normal; in particular, there were no cardiac arrhythmias or other conduction abnormalities. A brain MRI showed mild atrophy of the cerebellar vermis and hemispheres (Figure 1d,f) Overall, the ataxia was stable with mild improvement in motor skills.

### 2.2. Genetic Data

Two novel missense variants in *KCND3* were detected. The heterozygous c.1040C > G; p.(S347W) variant was found in the patient with adult-onset SCA19/22 (patient 1) while the c.1075T > G; p.(W359G) variant was present in the patient with congenital ataxia (patient 2). Segregation analysis demonstrated a de novo origin of the mutation in patient 2, while for patient 1 segregation could not be completed because the father was not alive. Both variants are predicted deleterious by in silico tools and have not been reported in available databases (i.e., dbSNP146, 1000 Genomes, ExAC and GnomAD). We classified the variants as pathogenic, according to ACMG parameters PVS1, PS2/PM6 and PP4 [9].

### 2.3. Molecular Localization of Disease-Associated K_V_4.3 Variants

**Figure 2:**
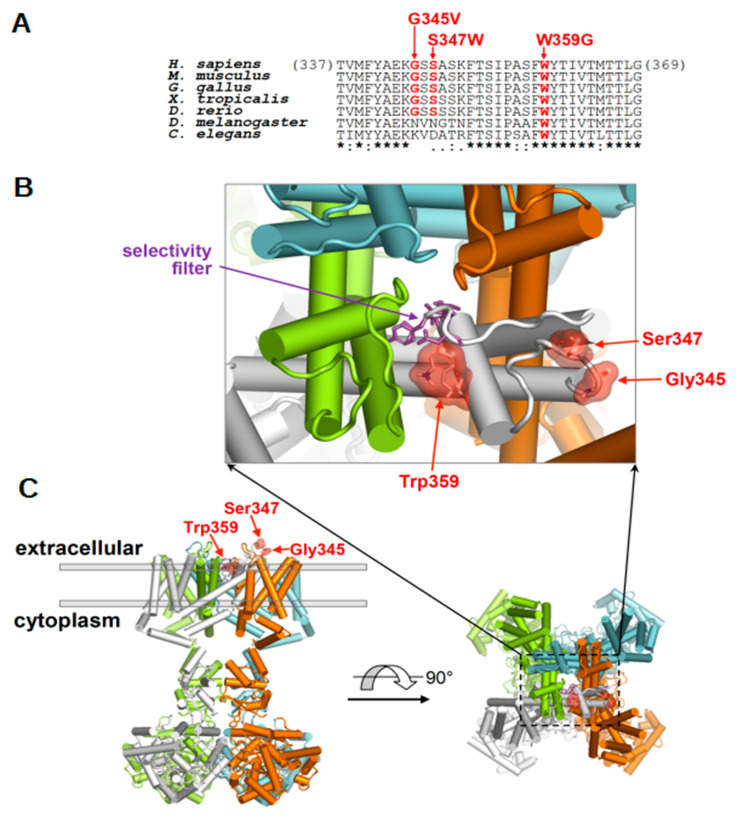
Topographic presentation and protein structure modeling for K_V_4.3 channel. (**A**) Amino acid sequent alignment of various K_V_4.3-relevant homolog and ortholog proteins. The residues G345, S347, and W359 (in red) are highly conserved across multiple animal species. (**B**,**C**) The homology models of the KCND3 protein are shown with the affected residues. The four identical monomers composing the KCND3 pore are shown in distinct colors. The selectivity filter (residues 367–372) is represented by magenta sticks (**B**). G345 and S347 are located proximal to the pore while W359 directly interacts with the selectivity filter.

Similar to other K_V_ channels, the K_V_4.3 subunit comprises six transmembrane segments (S1–S6) and an ion selectivity filter containing a pore loop in the S5–S6 linker region. The disease-related variants of p.G345V (a variant which was previously reported in a SCA19/22 family [2], but not functionally evaluated), p.S347W, and p.W359G are all located in the extracellular region of the S5–S6 linker close to the S5 transmembrane segment (Figure S1). The sequence alignment analysis shows that the G345 and S347 residues are well conserved among vertebrates while the W359 residue is a variant shown in worms. In particular, the p.G345V and p.S347W changes both modify a loop proximal to the pore while p.W359G directly alters one of the helices that form the pore and the selectivity filter (Figure 2). 

### 2.4. Altered Voltage Dependent Current Amplitudes of Disease-Associated K_V_4.3 Mutants 

**Figure 3:**
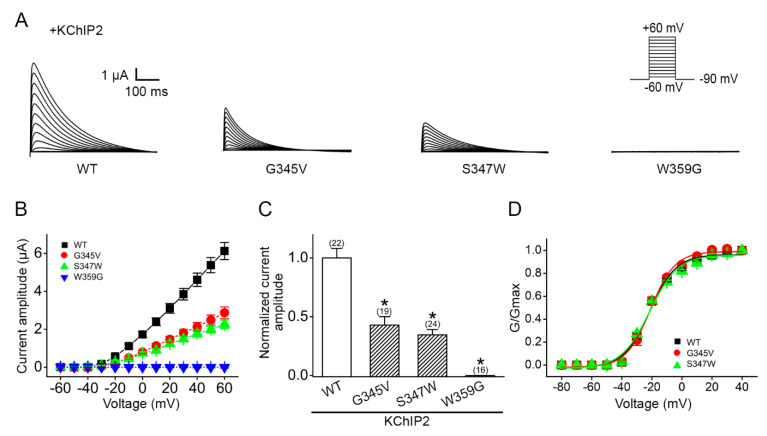
Loss-of-function channel phenotype of disease-related K_V_4.3 mutants. Functional expression of Myc-K_V_4.3 channel subunits in the presence of the auxiliary KChIP2 subunit in Xenopus oocytes. (**A**) Representative current traces in response to a voltage protocol comprising test potentials ranging from -60 mV to +60 mV in 10-mV steps. In contrast to the robust K+ currents observed in the WT, expression of individual K_V_4.3 p.G345V, p.S347W, and p.W359G mutant resulted in significant reduction in outward K+ currents. (**B**) Peak K+ current amplitudes are plotted against corresponding test pulse potentials (I-V curves). (**C**) Normalized peak current amplitudes at +60 mV: WT, 1.00 ± 0.10; p.G345V, 0.45 ± 0.05; p.S347W, 0.39 ± 0.05; p.W359G, 0.00 ± 0.00. Data from the same batch of oocytes on a given day were normalized with respect to the mean amplitude of the WT, followed by pooling multiple normalized data from different batches of oocytes. Numbers of observations are labeled in parentheses. Asterisks denote significant differences from the WT control (*, *p* < 0.05). (**D**) Steady-state activation curves of individual WT, p.G345V, and p.S347W K_V_4.3. Lines represent Boltzmann equation fits to the data points. The half-activation voltage (V0.5a) are: WT, −28.9 ± 0.5; p.G345V, −28.9 ± 0.5; p.S347W, −28.9 ± 0.5.

We ascertained the functional features of these disease-related mutants by performing a two-electrode voltage recording of K_V_4.3 currents in Xenopus oocytes. As demonstrated in Figure 3a, in the presence of the auxiliary subunit KChIP2, the expression of K_V_4.3 WT resulted in substantial K^+^ currents. The K_V_4.3 p.G345V and p.S347W mutants also generated functional K^+^ channels, whereas no discernible signal was detected for the p.W359G mutant (Figure 3A). Nonetheless, the K_V_4.3 p.G345V and p.S347W mutants exhibited notably decreased K^+^ current levels, leading to more than a 50% reduction in the current amplitude at +60 mV (Figure 3b,c). The steady-state voltage-dependent gating property of the p.G345V and p.S347W mutants, however, is comparable with that of K_V_4.3 WT, as indicated by the lack of a significant alteration in the half-activation voltage (V_0.5a_) and the activation slope factor (ka) (Figure 3d). Overall, these data support the idea that the three cerebellar ataxia-related K_V_4.3 mutants are associated with a loss-of-function phenotype.

### 2.5. Reduced Protein Expression of the Disease-Associated K_V_4.3 Mutants

**Figure 4:**
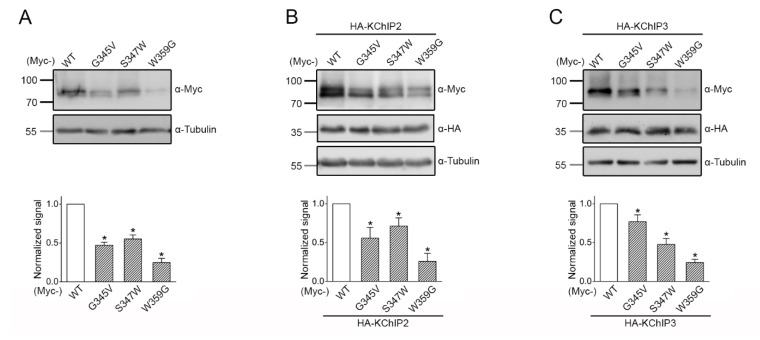
Disrupted proteostasis of the disease-associated K_V_4.3 mutants. Biochemical characterization of Myc-K_V_4.3 subunits in HEK293T cells. (Top panels) Representative immunoblots showing total protein expression of various K_V_4.3 constructs. Cell lysates were subject to immunoblotting analyses with the indicated antibodies (α-Myc, α-HA, and α-Tubulin). Molecular weight markers (in kDa) are labeled to the left. Tubulin expression was chosen as the loading control. (Bottom panels) Statistical analyses. Total K_V_4.3 protein density was standardized as the ratio to the cognate total tubulin signal, followed by normalization with respect to the WT control. (**A**) Myc-K_V_4.3 subunits were expressed alone (n = 6): WT, 1.00 ± 0.08; p.G345V, 0.47 ± 0.04; p.S347W, 0.55 ± 0.05; p.W359G, 0.25 ± 0.05. (**B**) Myc-K_V_4.3 were co-expressed with HA-KChIP2 (n = 6): WT, 1.00 ± 0.10; p.G345V, 0.56 ± 0.14; p.S347W, 0.71 ± 0.11; p.W359G, 0.26 ± 0.10. (**C**) Myc-K_V_4.3 were co-expressed with HA-KChIP3 (n = 14−18): WT, 1.00 ± 0.03; p.G345V, 0.77 ± 0.09; p.S347W, 0.48 ± 0.08; p.W359G, 0.24 ± 0.04. Asterisks denote significant difference from the WT control (*, *p* < 0.05).

Next, we studied K_V_4.3 protein expression in HEK293T cells. We began by examining the expression of the K_V_4.3 subunit in the absence of KChIP subunits. Figure 4a illustrates that, compared to their WT counterpart, the total protein level of p.G345V, p.S347W, and p.W359G significantly decreased by about 53%, 45%, and 75%, respectively. Upon co-expression with the auxiliary KChIP2 or KChIP3 subunits, which are known to promote K_V_4.3 protein expression and cell surface localization [10,11,12], the non-functional p.W359G mutant still exhibited more than a 70% reduction in total protein levels (Figure 4b,c). Likewise, in the presence of either auxiliary subunit, the protein expression of the functional p.G345V and p.S347W mutants remained notably decreased by about 23–52% (Figure 4b,c). Together, these biochemical observations suggest that the functional deficit of the K_V_4.3 mutants may, in part, be attributed to impaired proteostasis.

## 3. Discussion

Spinocerebellar ataxias (SCAs) are a group of rare, autosomal dominant diseases characterized by progressive ataxia and cerebellar degeneration, typically with onset in adulthood [13]. Nonprogressive congenital ataxia (NPCA) refers to children with early evidence of cerebellar ataxia, without clinical progression on follow-up, and a tendency towards gradual improvement. Neuroimaging usually reveals a progressive enlargement of cerebellar fissures resembling cerebellar atrophy, likely resulting from a combination of cerebellar hypoplasia and secondary atrophy. Signs of ataxia are always preceded by muscular hypotonia and delayed motor milestones and are usually not seen before the second or third year of life. Pathogenic missense variants in *CACNA1A*, *ITPR1* or *SPTBN2* are the most frequently identified causes of NPCA [14]. Only a few cases carrying de novo *KCND3* variants with developmental delay and congenital ataxia have been reported including a patient with early onset cerebellar ataxia complicated by intellectual disability, epilepsy, attention deficit hyperactivity disorder, strabismus, oral apraxia and joint hyperlaxity carrying a de novo p.R293_Phe295 duplication in the voltage-sensor domain (Figure S4) of the K_V_4.3 channel causing a severe shift in channel gating to more depolarized voltages [4]. In another patient presenting with developmental delay, congenital ataxia, and autistic spectrum disorder, a de novo p.G371R variant was identified but no functional studies were performed [5]. According to a recent review of 68 cases with *KCND3*-related neurological disorders, in the early-onset cohort (comprising 15 patients) intellectual disability and epilepsy were the most frequently presenting signs whereas ataxia had a less predictable course in terms of age of onset, severity and progression rate. A patient carrying a de novo p.Ser301Pro variant, presented neurodevelopmental delay and focal epilepsy but developed a complex movement disorder with cervical and upper limb dystonia and a mixed parkinsonian-ataxic gait in adulthood [15]. 

Human K_V_4.3 channels are critical for regulating neuronal excitability and action potential firing patterning [16,17]. In this study, we characterized the functional and biochemical phenotypes of two novel *KCND3* mutations, p.S347W (adult-onset ataxia) and p.W359G (harbored by the patient with congenital ataxia), as well as a previously reported adult-onset p.G345V variant [2]. As clearly delineated in Figure 3, the novel K_V_4.3 p.W359G mutant is virtually non-functional with an undetectable K^+^ current, while the p.G345V and the novel p.S347W mutant channels exhibit significantly reduced K^+^ currents. Moreover, the three disease-causing mutations lead to a substantial defect in K_V_4.3 protein expression (Figure 4). Therefore, similar to the other ataxia-related K_V_4.3 mutations [1,2,18,19,20,21], our findings support the idea that the p.G345V, p.W359G, and p.S347W mutations are associated with loss-of-function phenotypes. 

Including the two novel variants reported herein, currently there are approximately 20 *KCND3* variants associated with cerebellar ataxia (Figure S1) [1,2,18,19,20,21,22,23,24,25]. Interestingly, the majority of these ataxia-related mutations are localized in the transmembrane S5–S6 linker region of the human K_V_4.3 protein, which contributes to forming the pore of the K^+^ channel. Consistent with this notion, the three K_V_4.3 mutations studied in the current report also take place in the extracellular portion of the pore-forming S5–S6 linker; in particular, the p.W359G residue is directly in contact with the selectivity filter, which could explain the total absence of K^+^ recorded in this mutant (Figure 2). It is conceivable that these ataxia-related amino acid substitutions may result in a substantial structural alteration of the ion passage pore at the S5–S6 linker, but not the voltage-sensing transmembrane S4 region. In line with this idea, the functional K_V_4.3 p.G345V and p.S347W mutant channels display considerably reduced K^+^ current levels with no obvious alteration in the voltage-dependent gating properties (Figure 3). Furthermore, it is important to point out that the extent of the K_V_4.3 channel function loss appears to correlate with the complexity of neurological deficits observed in the patients. Specifically, the patients harboring the reduced-function p.G345V and p.S347W mutations are characterized by adult-onset pure cerebellar ataxia with no significant cognitive impairment. In contrast, the patient with the non-functional p.W359G mutation is associated with a wider range of neurological features including developmental delay, and cognitive dysfunction, although cerebellar ataxia is milder and does not progress. 

Interestingly, spinocerebellar ataxia type 13 (SCA13) caused by mutations in *KCNC3*/K_V_3.3, also exists in distinct forms which have been associated with a phenotypic spectrum that includes both non-progressive congenital-onset ataxia and (slowly) progressive infancy to adult-onset cerebellar ataxia. Using zebrafish, it was demonstrated that the early-onset mutation p.R423H dramatically and transiently increased Purkinje cell excitability, abolishing the K^+^ current amplitude, impaired dendritic branching and synaptogenesis, and caused rapid cell death during cerebellar development, while the adult-onset mutation p.R420H did not significantly alter basal tonic firing in Purkinje cells, but reduced excitability during evoked high-frequency spiking. Purkinje cells expressing the adult-onset mutation matured normally and did not degenerate during cerebellar development [26]. 

Further studies will be necessary to determine the effects of *KCND3* disease-causing mutations on neuronal excitability and survival in cerebellar neurons in vivo. The present study broadens the spectrum of the known ataxic phenotypes and identifies additional variants for *KCND3*-related disorders, outlining the importance of SCA gene screening in early-onset and congenital ataxia.

## 4. Patients and Methods

The patients were recruited at the Children’s Hospital B. Gesù and University Hospital A. Gemelli, Rome. Blood samples were obtained after written informed consent from all participating subjects. The patients underwent a detailed neurological and neuroradiological examination.

### 4.1. Genetic Testing 

The patients were included in a next-generation sequencing (NGS) panel of genes whose mutations are causative of various forms of cerebellar ataxias, including all known SCA genes. Genomic DNA was extracted from the peripheral blood of the patients and their parents by using a NucleoSpin tissue extraction kit (Macherey-Nagel, Düren, Germany). The panel was designed using Nextera technology on a MiSeq platform (Illumina, San Diego, CA, USA), following the manufacturer’s protocol, with expected coverage of 99% of the targeted genomic regions. Mapping of sequences against the hg19 reference genome was performed by Bowtie2. Bioinformatic tools HaplotypeCaller (GATK ver. 4.3) and ANNOVAR were used to call and annotate the variants, respectively. Variants were filtered to include only variants covered by at least 20 reads and with mapping quality values exceeding a Phred-score of 30. Variants were analyzed under presumed autosomal recessive, dominant or *de novo* inheritance models. Variants of the probands were filtered to retain all variants predicted to have functional impact (i.e., nonsynonymous variants and changes affecting splice sites) by the available bioinformatics tools including PolyPhen-2 (http://genetics.bwh.harvard.edu/pph2/ accessed on 22 July 2019), Sorting intolerant from tolerant (http://sift.jcvi.org/ accessed on 22 July 2019), Mutation Taster (http://www.mutationtaster.org/ accessed on 22 July 2019), Alamut (http://www.interactive-biosoftware.com/ accessed on 22 July 2019) and Combined annotation dependent depletion (http://cadd.gs.washington.edu/hom accessed on 22 July 2019). A list of the rare/private (gnomAD frequency <0.5%, population-matched in-house DB frequency <1%) variants predicted to have functional impact (CADD score >15) was created. Segregation was verified by Sanger sequencing in the families. Accession numbers are as follows: human *KCND3* mRNA: NM_004980.4; human KCND3 protein: NP_004971.2. 

### 4.2. Protein Homology Modeling

Homology modelling of the human potassium voltage-gated channel subfamily D member 3 (KCND3/K_V_4.3) was made with the SCWRL4 [27] using the Protein Data Bank (PDB) structure 2A79 representing a Shaker K_V_1.2 potassium channel- beta subunit complex as the template. Molecular graphics were made with PyMOL (http://www.pymol.org accessed on 2 December 2020).

### 4.3. Expression Plasmids

Amino-terminal Myc-tagged K_V_4.3 (Myc-K_V_4.3) was generated by subcloning human 4 K_V_4.3 cDNA into the pcDNA3.1-Myc vector (Invitrogen). Disease-associated K_V_4.3 mutant constructs were generated by using the QuikChange Site-Directed Mutagenesis Kit (Stratagene). Amino-terminal HA-tagged KChIP2 (HA-KChIP2) and KChIP3 (HA-KChIP3) were created by subcloning the cDNA for human KChIP2 (AF199598) and KChIP3 (AF199599), respectively, into the pcDNA3-HA vector (Invitrogen). All constructs were verified by DNA sequencing. For electrophysiological studies in *Xenopus* oocytes, appropriate restriction enzymes were employed for linearizing cDNAs, followed by in vitro transcription of capped cRNAs with the mMessage mMachine T7 kit (Ambion).

### 4.4. Electrophysiology

Adult female *Xenopus laevis* (African *Xenopus* Facility) were anesthetized by immersing in ice water containing Tricaine (1.5 g/L), followed by dissection for isolating ovarian follicles, which were then cut into pieces and incubated in ND96 [(in mM): 96 NaCl, 2 KCl, 1.8 MgCl2, 1.8 CaCl2, and 5 HEPES, pH 7.2]. To remove the follicular membrane, *Xenopus* oocytes were incubated in collagenase-containing (2 mg/mL) Ca^2+^-free ND96 solution on an orbital shaker (200 rpm) for 30–60 minutes at room temperature. The collagenase-free, Ca^2+^-free ND96 was then used to wash the oocytes for several times, and stage V–VI oocytes were selected for subsequent cRNA injection. cRNAs for individual K_V_4.3 and KChIP2 were mixed in an equimolar ratio and injected into oocytes in a total volume of 41.4 nL. Injected oocytes were incubated in ND96 at 16 °C for 2–3 days before being used for electrophysiology experiments. A conventional two-electrode voltage-clamp recording with an OC-725C oocyte clamp (Warner Instruments) was employed to record K^+^ currents through K_V_4.3 channels. The recording bath contained Ringer solution ((in mM): 3 KCl, 115 NaCl, 1.8 CaCl_2_, 10 HEPES, and 0.4 niflumic acid, pH 7.4 with methanesulfonic acid). With an agarose bridge, the bath solution was connected to a ground chamber (containing 3 M KCl), into which two ground electrodes were inserted. Borosilicate electrodes (0.1–1 MΩ) filled with 3 M KCl were used for current injection and voltage recording. Data were acquired and digitized via Digidata 1440A using pCLAMP 10.2 (Molecular Devices). Oocytes were held at −90 mV and leak currents arising from passive membrane properties were subtracted by using the −P/4 method provided in the pCLAMP system. Data were filtered at 1 kHz and digitized at 10 kHz, and all data were recorded at room temperature (20–22 °C). A previously reported data normalization procedure [9] was adopted to avoid potential biases imposed by channel expression variations among different batches of oocytes. To generate a peak K^+^ current-voltage (I–V) curve for studying the voltage-dependent activation property of K_V_4.3, oocytes were held in a −90 mV membrane potential followed by being subjected to 500-ms test pulses, ranging from −60 mV to +60 mV in 10 mV increments. From the peak I–V curve, the relative K^+^ channel conductance (G/Gmax) at a given test potential was calculated as G/G_max_ = ∆I_K_/∆I_K,+60_, where ∆I_K_ is the current increment determined from the peak K+ current difference between adjacent test pulses, and ∆I_K,+60_ is the ∆I_K_ at the test potential +60 mV. Steady-state voltage-dependent activation of K_V_4.3 was then analyzed by fitting a G/Gmax-voltage (G-V) curve with a Boltzmann equation: G/G_max_ = 1/{1 + exp[(V_0.5a_−V)/*k_a_*]}, where V_0.5a_ is the half-activation voltage, and *ka* is the activation slope factor. 

### 4.5. Cell Culture and Transfection

Human embryonic kidney (HEK) 293T cells were maintained in DMEM supplemented with 2 mM L-glutamine, 10% fetal bovine serum (Hyclone), and 100 units/mL penicillin/streptomycin with 95% air and 5% CO_2_ in a humidified incubator at 37 °C and passaged every 3–4 days. Cells were plated onto 6-, 12- or 24-well cell culture plates 24 h before transfection. Transient transfection was performed by using a standard calcium phosphate method. Unless stated otherwise, 0.5–1 μg of K_V_4.3 cDNA was added into each well. For co-expression experiments, cDNAs for WT or individual mutant K_V_4.3 and the auxiliary subunit (KChIP2 or KChIP3) were mixed in a molar ratio of 1:1. Transfected cells were maintained at 37 °C for 48 h before being processed for further experiments.

### 4.6. Immunoblotting 

Transfected cells were washed twice with ice-cold PBS ((in mM): 136 NaCl, 2.5 KCl, 1.5 KH_2_PO_4_, and 6.5 Na_2_HPO_4_, pH 7.4), centrifuged, and resuspended in lysis buffer ((in mM): 150 NaCl, 5 EDTA, 50 Tris-HCl, 1 PMSF, 1% Triton X-100, pH 7.6, supplemented protease inhibitor mixture). Laemmli sample buffer was added to the lysates, and samples were sonicated on ice (3 times for 5 s each) and heated at 70 °C for 5 min. Samples were then separated by 7.5–10% SDS-PAGE, electrophoretically transferred to nitrocellulose membranes, and detected using mouse anti-Myc (1:5000; clone 9E10), rat anti-HA (1:5000; Roche), or rabbit anti-α-tubulin (1:5000; GeneTex). Blots were exposed to horseradish-peroxidase-conjugated goat anti-mouse IgG (1:5000; Jackson ImmunoResearch), goat anti-rabbit IgG (1:5000; Jackson ImmunoResearch), or goat anti-rat IgG (1:5000; Santa Cruz Biotechnology) and revealed by an enhanced chemiluminescence detection system (Thermo Scientific). Densitometric scans of immunoblots were quantified with ImageJ (National Institute of Health). Data shown are representative of at least three independent experiments.

### 4.7. Statistical Analyses

All statistical analyses were performed with Origin 7.0 (Microcal Software). Numerical values were presented as mean ± SEM. The significance of the difference between two means was tested using Student’s *t*-test. Where necessary, means from multiple groups were additionally compared using one-way ANOVA.

## Figures and Tables

**Table 1:** Clinical overview of individuals harboring the *KCND3* variants analyzed in this study.

Patient	Family C (Lee et al. 2012)	1	2
Genetic variant	c.1034G > T	c.1040C > G	c.1075T > G
Protein	p.G345V	p.S347W	p.W359G
Inheritance	AD	n.d	de novo
Incomplete penetrance	Yes	-	-
Onset	35–55 y	45 y	1.5 y
First symptom	Gait disorder	Gait disorder	Hypotonia, DD
Ataxia	Yes	Yes	Yes
Nystagmus	No	No	No
Dysarthria	Yes	Yes	Yes
Saccadic pursuit	Yes (1/3)	No	Yes
Cognitive delay	No	No	Yes
Developmental delay	No	No	Yes
Paroxysmal features	No	No	No
Movement disorders	No	No	No
Pyramidal signs	Yes (1/3)	No	No
Seizures Clinical course Brain MRI	No Slowly progressive CA	No Slowly progressive CA	No stable mild CA

Abbreviations: DD = developmental delay; AD = autosomal dominant; CA = cerebellar atrophy.

## Data Availability

The data that support the findings of this study are available from the corresponding authors, upon reasonable request.

## Supplementary Materials

The following are available online at https://www.mdpi.com/1422-0067/22/9/4986/s1, Figure S1: Schematic representation of the KCND3 protein (NP_004971.2) with all previously identified variants, including *KCND3*-related cardiac phenotypes or Brugada syndrome (MIM#616399). Video S1: Video of patient 1 documenting the cerebellar syndrome: ataxia and incoordination but not gaze palsy or nystagmus.
