# Supplementary figures and images for: Novel KCND3 Variant Underlying Nonprogressive Congenital Ataxia or SCA19/22 Disrupt KV4.3 Protein Expression and K+ Currents with Variable Effects on Channel Properties
Source: Int J Mol Sci. 2021 May 7;22(9):4986. doi: 10.3390/ijms22094986 (PMC8125845; doi:10.3390/ijms22094986)

**Legend:**

NEUROLOGICAL phenotypes

CARDIONEUROLOGICAL phenotypes

CARDIAC phenotype

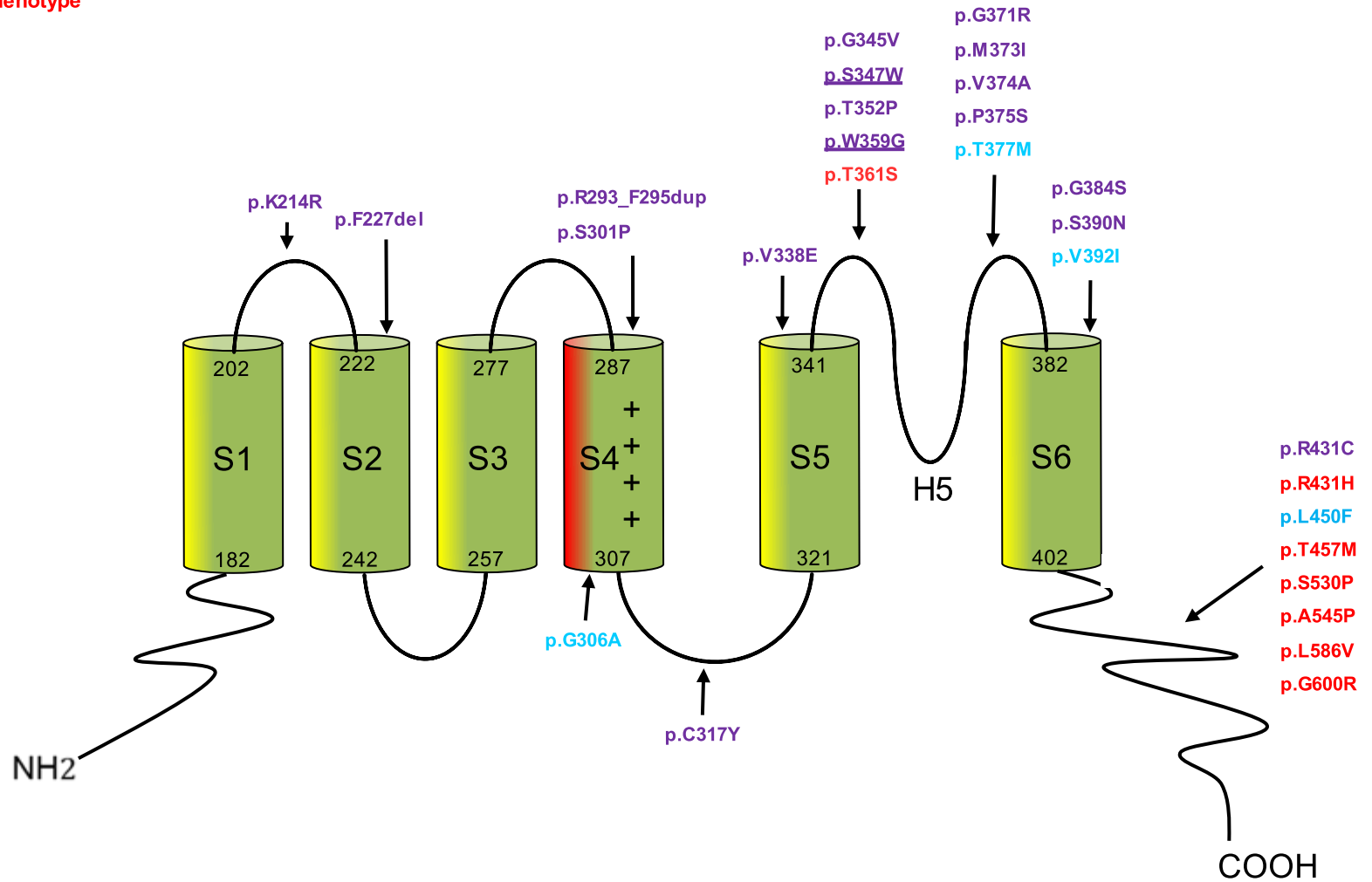

Supplement: Supplementary file 1 [file ijms-22-04986-s001.zip › Supps/FIGURE S1.pdf]
